# Physiotherapist as primary assessor for patients with suspected knee osteoarthritis in primary care—a randomised controlled pragmatic study

**DOI:** 10.1186/s12891-019-2690-1

**Published:** 2019-07-13

**Authors:** Chan-Mei Ho, Carina A. Thorstensson, Lena Nordeman

**Affiliations:** 1 Region Västra Götaland, Närhälsan Health Unit, Primary Health Care, Lidköping, Sweden; 2 Department of Health and Rehabilitation, Unit of Physiotherapy, University of Gothenburg, Sahlgrenska Academy, Institute of Neuroscience and Physiology, Gothenburg, Sweden; 3 Department of Neurobiology, Care sciences and Society, Unit of Physiotherapy, Karolinska Institutet, Stockholm, Sweden; 4 Region Västra Götaland, Närhälsan, Research and Development Primary Health Care, Research and Development Center Södra Älvsborg, Borås, Sweden

**Keywords:** Delivery of health care, Disease management, Treatment outcome, Quality of life, Osteoarthritis, Knee

## Abstract

**Background:**

In Swedish primary care, the healthcare process for patients with knee osteoarthritis (KOA) can be initiated by a physician or physiotherapist assessment. However, it is unclear how the different assessments affect the healthcare processes and patient reported outcomes over time. The purpose of this study was to examine the differences in health-related quality of life (HrQoL), adjusted for pain and physical function, for patients with KOA when the healthcare process is initiated by a physiotherapist assessment compared to a physician assessment in primary care.

**Methods:**

An assessor-blinded randomised controlled pragmatic trial. Using a computer-generated list of random numbers, patients seeking primary care during 2013–2017 with suspected KOA were randomised to either a physiotherapist or physician for primary assessment and treatment. Data was collected before randomisation and at 3, 6, and 12-month follow-ups. Primary outcome was HrQoL using EuroQol 5 dimensions 3 levels questionnaire, index (EQ-5D-3L index) and a visual analogue scale (VAS) (EQ-5D-3L VAS); pain intensity was measured with VAS (0–100) and physical function measured with the 30-s chair stand test. Mixed effect model analyses compared repeated measures of HrQoL between groups. The significance level was *p* < 0.05 and data was applied with intention-to-treat.

**Results:**

Patients were randomised to either a physiotherapist (*n* = 35) or physician (*n* = 34) for primary assessment. All 69 patients were included in the analyses. There were no significant differences in HrQoL for patients assessed by a physiotherapist or a physician as primary assessor (EQ-5D-3L index, *p* = 0.18; EQ-5D-3L VAS, *p* = 0.49). We found that HrQoL changed significantly 12 months after baseline assessment for all patients regardless of assessor (EQ-5D-3L index, *p* < 0.001; EQ-5D-3 L VAS, *p* = 0.0049). No adverse events or side effects were reported.

**Conclusions:**

There were no differences in HrQoL, when adjusted for pain and physical function, for patients with KOA when the healthcare process was initiated with physiotherapist assessment compared to physician assessment in primary care. Both assessments resulted in significantly higher HrQoL at the 12-month follow-up. The results imply that physiotherapists and physicians in primary care are equally qualified as primary assessors.

**Trial registration:**

Retrospectively registered at http://clinicaltrial.gov, ID: NCT03715764.

**Electronic supplementary material:**

The online version of this article (10.1186/s12891-019-2690-1) contains supplementary material, which is available to authorized users.

## Background

Osteoarthritis (OA) is one of the most common joint diseases and a major cause of chronic musculoskeletal pain and disability in working and older adults [1, 2]. In Sweden, 14% of those over 45 are estimated having knee OA (KOA) [3]. Common OA symptoms are pain, morning stiffness, reduced range of motion, joint instability, swelling, muscle weakness and fatigue [4]. This directly affects patients’ social interactions, mental functioning, and sleep quality [5], and patients with KOA report among the lowest health-related quality of life (HrQoL) compared with patients suffering other chronic diseases [6]. This patient group has a twofold risk for sick leave, and the diagnosis entails a 40–50% higher risk for disability pension. KOA accounts for 2% of all sick days in Sweden [2]. Patients with OA are less active and have more comorbidities than the overall population [7]. OA causes activity limitations, especially in walking [8]. Walking disability is related to a greater risk of mortality [9], which is largely explained by lack of physical activity [10–12].

Over the last 40 years, the proportion of overweight and obesity in the Swedish population have increased from 35% to 56% among men, and 27% to 41% among women [13]. This will probably affect the incidence of OA since overweight is a strong risk factor [14]. Consultations to healthcare are expected to increase by 30–50% among patients with OA over the next 10 years [15] and primary care physicians will probably face this predicted escalation in OA consultations [16].

Early access to a physiotherapist (PT) has previously been shown safe and effective for patients with musculoskeletal disorders [17, 18]. A pilot study showed that most patients assessed by a rehabilitation professional first (PT, occupational therapist, psychologist or counsellor) did not need to see a physician later [19]. PTs as primary assessors reduce referrals, sick leave, and prescriptions of analgesics for most musculoskeletal conditions [20]. Previous studies of back and neck pain have shown that the most common expectation when consulting a clinician (PT or physician) is not recovery, but having their diagnosis confirmed [21, 22], which is similar to what has been seen in patients with OA [23]. At the same time, patients with OA seem reluctant to seek professional help, partly because they wait until their problems affect their lifestyle or safety (e.g. risk of falling) [23, 24]. Later in the healthcare process, patients with OA feel unsure when to see their physician, they believe that physicians were more for initial diagnosis rather than following treatment [25–27].

Imaging is not required to diagnose a typical presentation of OA (i.e. usage-related pain, short duration morning stiffness, age > 40, symptoms affecting one or more joints) [28]. Detectable radiographic changes are not always present in early OA [29, 30]. When imaging is not required to diagnose typical symptomatic OA, both physicians and PTs can act as primary assessors. European League Against Rheumatism (EULAR) guidelines do not recommend any particular healthcare provider for the initial assessment since evidence of the effectiveness of various forms of assessment is lacking. The recommendation is that the initial assessment should use a biopsychosocial approach including physical status, activities of daily living, participation in work, leisure or education, mood and health education needs, health beliefs and motivation to self-manage [31]. Core treatment of OA should be individualized and include patient education, an exercise regimen, weight loss if overweight or obese, reduction of adverse mechanical factors, and consideration of walking aids [31, 32]. Muscle strengthening exercises and maintaining physical activities, give patients with OA a better chance to maintain their level of physical function [33]. Physical activity interventions should be delivered by healthcare providers competent in treating this patient group [34]. Advice on exercise and pain relief comprises the bulk of the PT assessment, in comparison with other medical staff members [35–37], providing a key role in the acute and long-term management of OA. Common PT management in Sweden includes a nationwide program called “Better management of patients with OA” (BOA), consisting of patient education and supervised exercise to increase patients’ efficacy to self-manage the disease and increase their level of physical activity [38]. Participation in the Swedish BOA results in improvements in HrQoL, pain, and self-efficacy [39].

Management of expected increases in OA consultations by early referrals of patients with suspected OA to a PT could save time for primary care physicians and lead to fewer healthcare visits for patients. Early contact with a PT would also aid in assuring correct management through information about the disease and long-term guided strength training, physical performance and fitness. Today, in Swedish primary care, patients can access a PT without referral. Thus, patients with suspected KOA could have a first assessment by either a physician or a PT. However, it is unclear if there are differences between managements reflected in HrQoL, pain and physical function. The purpose of this study was to examine the differences in HrQoL, adjusted for pain and physical function, for patients with KOA when the healthcare process is initiated with PT assessment compared to a physician assessment in primary care. We hypothesise that all patients with suspected KOA could be assessed initially by a PT in primary care, and then referred to a physician if required.

## Methods

### Study design

**Fig. 1:**
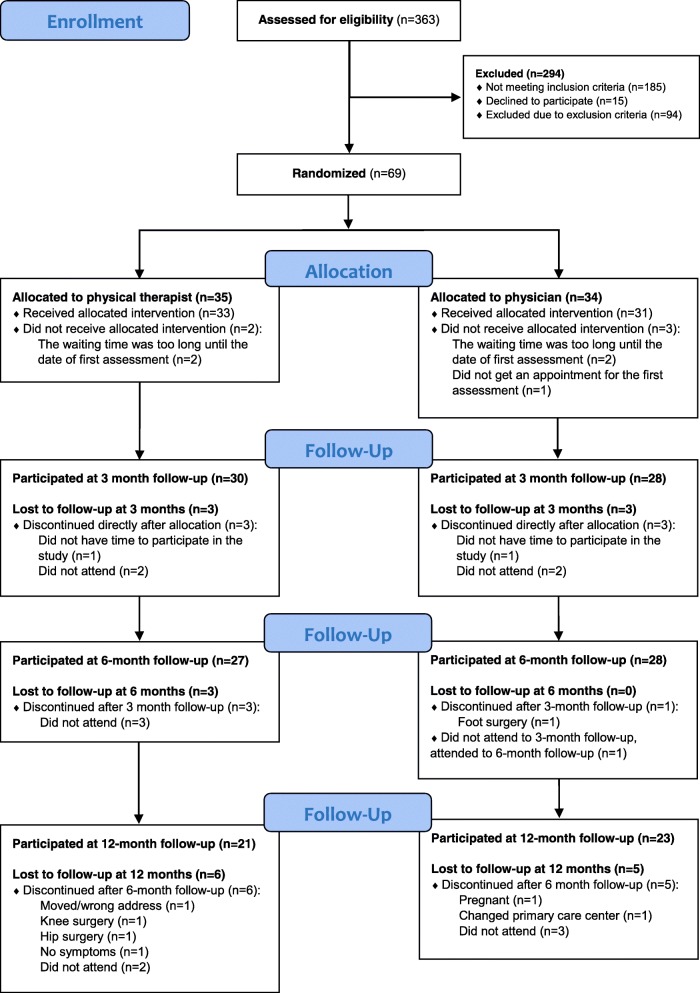
The participant flow

This is a multicentre, assessor-blinded, randomised controlled pragmatic trial comparing primary assessment, diagnosis, and treatment either by a PT or physician in primary care. The study comprised a healthcare process initiated either by a PT or physician assessment. Measurements were taken before randomisation (baseline) and at the 3-, 6- and 12-month follow-ups. The participant flow is illustrated in Fig. 1. The Regional Ethical Review Board in Gothenburg approved the study (reference number: 979–12). The study was retrospectively registered at clinicaltrial.gov, ID: NCT03715764.

### Participants

Participants were recruited from primary care centres and rehabilitation centres in primary care in southwestern Sweden from April 2013 to November 2017. There were only three recruiting primary care centres at the beginning of the trial, which appeared insufficient. One clinic withdrew due to organisational issues. It was decided to add more clinics to intensify patient inflow. Total recruiting units: 6 primary care centres and 3 rehabilitation centres in primary care. Inclusion criteria according to American College of Rheumatologys (ACR) clinical criteria, which were: age ≥ 38 years, knee pain most days of the past month, morning stiffness ≤30 min, and crepitus during active motion [40]. Exclusion criteria were knee pain due to trauma (i.e. not insidious debut), other diseases that could affect outcome measures (rheumatic or systemic diseases, severe somatic or mental diseases such as depression), pregnancy, or if already diagnosed or assessed by another healthcare provider due to current knee pain. The patient had to know enough Swedish to understand test instructions and complete self-administered questionnaires. The screening procedure was modified after 20 patients to intensify patient inflow. Morning stiffness and crepitus on active motion were removed from the inclusion criteria. Nurses screened for eligible participants at the primary care centres, and receptionists at the rehabilitation centres in primary care. All participants received oral and written information about the study, and provided written informed consent.

### Sample size

To detect a minimal clinical improvement in HrQoL of 0.121(SD 0.2) on the EuroQol 5 dimensions 3 level questionnaire (EQ-5D-3L) index [41, 42], with a two-sided 5% significance level and a power of 80%, a sample size of 50 patients per group was found necessary, given an anticipated dropout rate of 14%.

### Randomisation

Using a computer-generated list of random numbers, participants were randomly assigned to initial assessment, diagnosis and treatment by either a PT or physician. Each primary care centre (*n* = 6) consisted of 6–11 physicians and the rehabilitation centres in primary care (*n* = 3) had 3–10 PTs. One project coordinator was included from among healthcare providers in the study, but was neither involved in the screening procedures nor data collection. The project coordinator managed the sequence generation, allocation concealment, enrolment and assignments of participants, and kept the concealed randomisation scheme and sequentially numbered, sealed envelopes in a locked cupboard (in the same building as enrolment), available only to the project coordinator. The project coordinator revealed allocation to the participant and healthcare providers shortly after baseline measurement. Participants and healthcare providers in both groups were aware of the allocated group, whereas the data collector (CH), data analyst (CH) and statistician were blinded to allocation until completion of all outcome assessments. CH was not involved in assessing, diagnosing, or treating patients with KOA while the study was in progress.

### Interventions

Patients were allocated to either a PT or physician for initial assessment, diagnosis and treatment. The assessments or treatments by either PT or physician were conducted in accordance with Swedish treatment guidelines [43] and could vary depending on the patients’ symptoms. PT treatment could involve individual and/or group treatment. Individual treatment could include exercise regimen (PT led or home exercising), education, pain relief or walking aids. Group treatment included patient education and individualized exercise regimen, according to the BOA program [38]. BOA consisted of individual assessment, patient education (3 sessions), and six weeks of exercising (PT led or home exercising). Physician treatment could include prescriptions, referrals to x-ray examination, a PT, or another healthcare provider. With the purpose to examine daily clinical setting, patients could see the other healthcare provider at any time after the first assessment if needed. Consultations with other healthcare providers were registered between baseline and the 12-month follow-up. This data will be presented in a cost-efficiency study, registered at clinicaltrial.gov, ID: NCT03822533.

### Outcome measures

The primary outcome measure was HrQoL, using EQ-5D-3L [41, 44, 45]. The questionnaire contains five dimensions: mobility, self-care, usual activities, pain/discomfort and anxiety/depression. For each dimension, the patient can choose between three levels best describing how they experience their state of health on the day of measurement (no problems = level 1, some problems = level 2, or extreme problems = level 3). The result of the questionnaire is an index on a scale between 1 and − 0.594 calculated using the United Kingdom’s value sets [46]. An index of 1 indicates full health. The EQ-5D-3L includes a visual analogue scale (EQ-5D-3L VAS), where the patient marks on a scale describing their health state on the day of measurement. The scale ranges from 0 to 100, where 0 is the worst imaginable health state and 100 is the best imaginable health state [42, 44, 47]. EQ-5D-3L has shown good test-retest reliability and validity for patients with KOA [47].

Demographic data was collected at baseline, including age, sex, national origin, social status, level of education, employment, pain duration, and height and weight to calculate Body Mass Index (BMI). Pain intensity over the past month was measured by a visual analogue scale (VAS) [48] which ranged from 0 to 100; extreme points 0 and 100 were anchored with no pain and worst imaginable pain, respectively, 1–20 was anchored with light pain, 21–40 moderate pain, 41–60 moderately severe pain, 61–80 severe pain, and 81–99 unbearable pain. Physical function was measured using the 30-s Chair Stand Test (30CST) [49]. The score was the total number of stands executed correctly from sitting on a chair within 30 s (more than halfway up at the end of 30 s was considered a full stand). Incorrectly executed stands (incomplete stands, or not seated between the stands) were not counted.

### Statistical analysis

Demographic data was analysed descriptively and presented as numbers and per cent, mean and standard deviation, and median and 25th to 75th percentiles. Mixed effect model analyses were used to compare the repeated measures of HrQoL between groups (EQ-5D-3L index and EQ-5D-3L VAS). Independent variables in the model were checked for collinearity using Spearman’s rank correlation coefficient (r ≤ 0.7), boxplot overlap, and cross tables (for > 80% observations in diagonal and cells > 5 observations). The mixed effect model analysis consisted of two models, Model 1 and Model 2. Model 1 (unadjusted): Variables: Group, Time and Group*Time (interaction between Group intervention and Time). Model 2 (final model with confounder adjustment): Based on Model 1, with confounder adjustment according to the criteria described below. Variables considered to be possible confounders were age, sex, body mass index (BMI), educational level, pain intensity and physical function. Possible confounders were added one at a time to Model 1, and carried forward to the final model if *p* < 0.20. Variables (Group, Time ad Group*Time) in the final models were considered statistically significant if *p* < 0.05. The model means will be presented in graphs to illustrate the direction of change in HrQoL and how the curves change during a 12-month period of time. If differences were found between the groups’ curves, additional analyses were made to examine possible significant differences in parts of the healthcare process. Data was analysed statistically in the Statistical Package for Social Science for Windows 22.0 [50]. Data was applied with intention-to-treat where patients received the randomised allocated intervention, i.e. the first assessment either by a PT or physician.

## Results

To establish if HrQoL differed between the effects of being assessed by a PT or a physician for suspected KOA, 69 patients were randomised to either a PT or physician as primary assessor. Most of the patients, 79%, participated in the 6-month follow up and 64% completed the 12-month follow-up.

Demographic data and clinical characteristics are presented in Table 1. All 69 patients were included in the mixed effect model analyses. We found that HrQoL improved significantly 12 months after assessment for all patients regardless of assessor (variable “Time”: EQ-5D-3L index, *p* < 0.001; EQ-5D-3L VAS, *p* = 0.0049). There were no significant differences in HrQoL between PTs and physicians as primary assessors (variable “Time*Group”: EQ-5D-3 L index, *p* = 0.18; EQ-5D-3 L VAS, *p* = 0.49). See Table 2 for EQ-5D-3L index results and Table 3 for EQ-5D-3L VAS results. The final model of the EQ-5D-3L index was adjusted for the confounder’s sex, educational level, pain intensity and physical function. The final model of EQ-5D-3 L VAS was adjusted for the confounder’s sex, pain intensity and physical function.

**Table 1:** Demographic features of the groups at baseline assessment

	Physical therapy assessment (*n* = 35)	Physician assessment (n = 34)
	Mean (SD); median [25th to 75th percentile] or % (n)	Mean (SD); median [25th to 75th percentile] or % (n)
Age (years)	62 (11.6); 63 [52–71]	59 (11.5); 57 [48–68]
Sex (female)	60% (21/35)	68% (23/34)
Origin		
Born in Sweden	86% (30/35)	94% (32/34)
Social status		
Partner/Married	89% (31/35)	77% (26/34)
Level of education		
Primary school (≤ 9 years)	23% (8/35)	12% (4/34)
Secondary school (10–12 years)	43% (15/35)	59% (20/34)
Tertiary school (> 12 years)	34% (12/35)	29% (10/34)
Current employment		
Employed/working	54% (19/35)	50% (17/34)
Working rate (%)	88 (4.7); 100 [81–100]	93 (4.2); 100 [100–100]
Unemployed	0% (0/35)	3% (1/34)
Retired/early retirement	43% (15/35)	38% (13/34)
Sick leave	3% (1/35)	6% (2/34)
Pain duration (months)	14 (22); 9 [3–12]	10 (16); 4 [2–11]
BMI^a^ (kg/m^2^)	30 (4.4); 29 [26–31]	29 (6.7); 27 [25–31]
BMI: normal weight (18,5-24,9)	9% (3/35)	29% (10/34)
BMI: overweight (25–29,9)	54% (19/35)	38% (13/34)
BMI: obese (> 30)	37% (13/35)	32% (11/34)
HrQoL^b^ (EQ-5D-3L)		
Index	0.73 (0.121); 0.73 [0.69–0.80]	0.62 (0.222); 0.73 [0.62–0.73]
VAS (0–100)	73 (17.5); 80 [68–90]	68 (21.1), 70 [54–89]
Pain intensity (VAS 0-100)	45 (15.9); 47 [35–55]	52 (16.4); 51 [40–69]
Physical function (30CST)^c^	12 (4.6); 12 [9–14]	11 (3.3); 11 [8–13]

^a^Body Mass Index;

^b^Health-related Quality of Life. Higher values indicate better health-related quality of life

^c^30-s Chair Stand Test. Higher values indicate better function

**Table 2:**
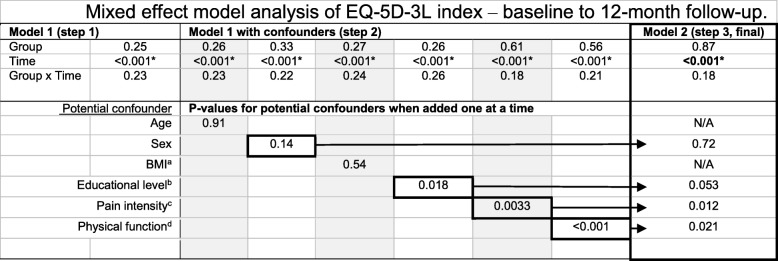
Mixed effect model analysis of EQ-5D-3L index – baseline to 12-month follow-up

Presenting *p*-values from regression analyses using mixed effect models

Model 1: Model included the variables Group, Time and Group x Time

Model 1 with confounder: Confounders were added separately to Model 1. Confounding variables with *p*-values < 0.2 were carried forward to the final model

Model 2: Final model, adjusted for confounders (sex, educational level, pain intensity and physical function)

Group: PT group resp. physician group

Time: Measurements at baseline, 3-, 6- and 12-month follow-ups

Group x Time: Statistical interaction of group and time

^a^Body Mass Index

^b^Educational level, dichotomized variables - primary and secondary or tertiary school

^c^Pain intensity, VAS 0–100

^d^Physical function, 30-s Chair Stand Test

*Statistically significant, *p* < 0.05

**Table 3:**
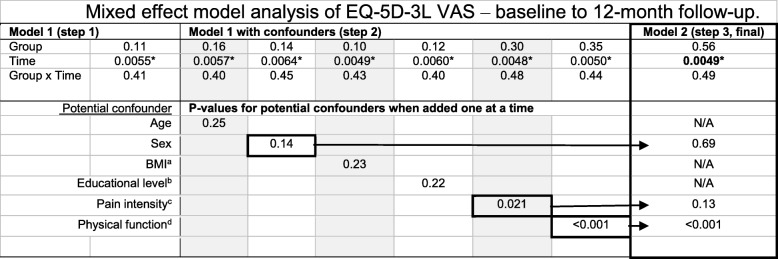
Mixed effect model analysis of EQ-5D-3L VAS – baseline to 12-month follow-up

Presenting *p*-values from regression analyses using mixed effect models

Model 1: Model included the variables Group, Time and Group x Time

Model 1 with confounder: Confounders were added separately to Model 1. Confounding variables with p-values < 0.2 were carried forward to the final model

Model 2: Final model, adjusted for confounders (sex, pain intensity and physical function)

Group: PT group resp. physician group

Time: Measurements at baseline, 3-, 6- and 12-month follow-ups

Group x Time: Statistical interaction of group and time

^a^Body Mass Index

^b^Educational level, dichotomized variables - primary and secondary or tertiary school

^c^Pain intensity, VAS 0–100

^d^Physical function, 30-s Chair Stand Test

*Statistically significant, *p* < 0.05

**Fig. 2:**
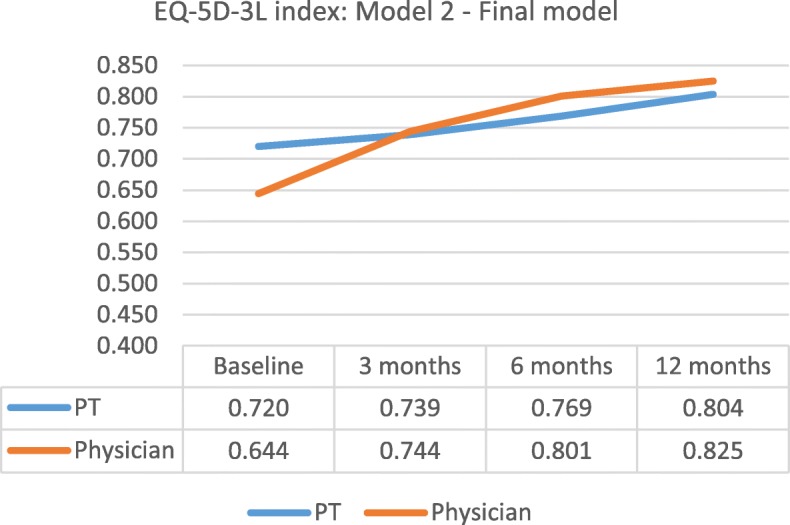
Model means for EQ-5D-3L index, Model 2: adjusted model using confounders

**Fig. 3:**
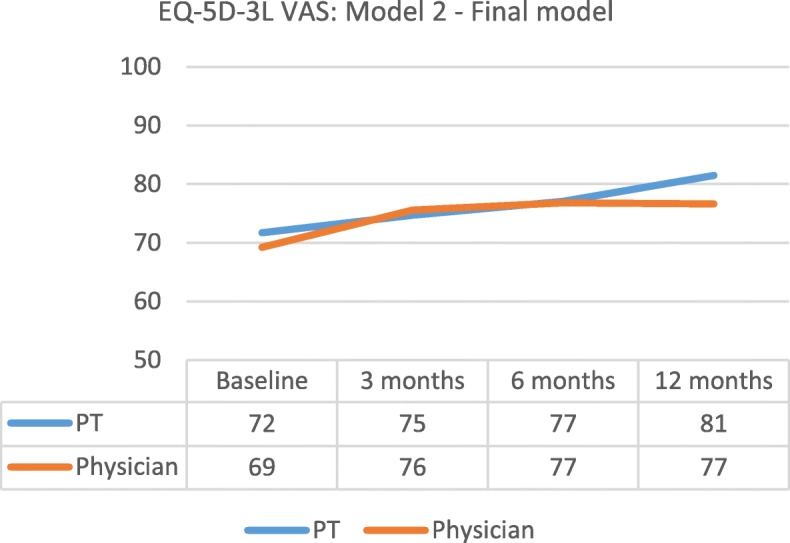
Model means for EQ-5D-3L VAS, Model 2: adjusted model using confounders

The model means for the EQ-5D-3L index increased for both groups. The physician group had a larger increase from baseline to the 12-month follow-up (PT = + 0.084, physician = + 0.181). For the EQ-5D-3L VAS, the total increase from baseline to 12-month follow-up in model means was similar between groups (PT = + 9, physician = + 8). The changes in model means are illustrated in Figs. 2 and 3 for adjusted models.

The model means showed an increase in EQ-5D-3L index for the physician group only for the period between baseline and the 3-month follow-up. Additional mixed effect model analyses were made for the first three months only. The final model showed no significant change in HrQoL for both groups (variable “Time”: EQ-5D-3L index, *p* = 0.42; EQ-5D-3L VAS, *p* = 0.99), or significant differences between PTs and physicians as primary assessors (variable “Time*Group”: EQ-5D-3L index *p* = 0.24; EQ-5D-3L VAS, *p* = 0.55). See Additional file 1 a and b.

## Discussion

The findings of this study imply that PTs can be the first assessor when patients with suspected KOA seek primary care for the first time. Our results are similar to previous findings showing that PTs are appropriate primary assessors for patients with musculoskeletal disorders [19, 20, 51]. PT as primary assessor is suggested as a model that uses healthcare resources more efficiently where the most appropriate healthcare professional assesses the patient’s needs [52]. Several studies [18, 53–57] have reported that patients experienced as much or even greater patient satisfaction with a PT assessment than with physician assessment. Thus, using this model of care could require recruitment of more PTs, which could mean increased costs. Future research is needed to explore how patients with KOA experience PTs as primary assessors and the cost efficiency of this task-shifting model.

In this study, we found no statistically significant differences in HrQoL between groups 12 months after assessment. Even though the physician group had a 0.097 larger increase in model means for the EQ-5D-3L index 12 months after assessment, this does not exceed the minimal clinical difference of 0.121. The reason for the large improvement in HrQoL for the physician group over the first three months could be related to the baseline mean value for the EQ-5D-3L index, which was much lower for the physician group when compared with the PT group. Patients allocated to a PT as primary assessor were somewhat older and had a slightly higher BMI at baseline. These patients also had longer pain duration, but graded lower pain intensity and had better physical function, which could also explain why patients in the PT group rated a higher HrQoL. The variation in the baseline values of EQ-5D-3L index were adjusted for in the mixed effect model analyses where patterns of change for each patient were used in the analysis [58]. The pragmatic study design might have contributed to a larger variation in patient characteristics within groups [59]. A larger sample size and/or use of a questionnaire with more levels such as EQ-5D-5L [60], could probably provide a better distribution of the EQ-5D index values at baseline.

Both groups improved in HrQoL 12 months after assessment. PT treatment in this study (i.e. the BOA program) were individualized, comprising patient education and exercise regime with the purpose of increasing patients’ abilities to self-manage. This program has been developed in accordance with national and international guidelines [31, 32]. Participation in the BOA program decreases pain, and increases HrQoL and self-efficacy [39]. Exercise therapy, with or without being combined with other treatments, is an effective intervention to improve HrQoL in patients with KOA [61, 62]. It is possible that most patients in the present study received PT treatment, either by randomisation to a PT as primary assessor or were referred to a PT by a physician. This could explain why the improvements in HrQoL were seen in both groups. If there were no significant differences in HrQoL regardless of the primary assessor, and most patients probably ended up with PT treatment, one way to make the healthcare process more efficient for patients with KOA could involve PTs as the sole primary assessor and use physicians when required.

Most patients were recruited from primary care centres, implying that they sought a physician consultation for their knee pain. It might be possible that the patients in the physician group were positively affected by the fact that they met a physician, which they expected from the beginning. Patients expect investigations such as x-rays or magnetic resonance imaging (MRI) to provide evidence of their experienced problem. Lack of these investigations could be experienced as a possible barrier for being understood and helped [63]. In this study, it could have led to higher satisfaction in the physician group due to receiving expected examinations and treatments, which could have affected the results with a higher HrQoL, less pain and improved physical function because of decreased anxiety for their problems. It would have been interesting if the patients did not know what profession the primary assessor had in order to rule out a potential placebo effect. Unfortunately, this was not possible in this primary care setting because most primary care centres and rehabilitation centres in primary care have different locations.

Changing the inclusion criteria is one of the methodological limitations of this study. With two criteria removed, only age > 38 years and knee pain without traumatic onset was used to detect suspected KOA. Wesseling et al. have been using knee pain and age over 45 as the only inclusion criteria when screening for early OA. None of the participants in their study had radiographic signs for OA, and 76% fulfilled the clinical ACR criteria for KOA [64]. Another study [30] using only knee pain of less than three months’ duration, age 35–54 years, and no history of previous knee injury or inflammatory joint disease found that 70% had clinically classified OA according to ACR [40]. In the same study, they found that 86% of the patients with normal radiographs at baseline, later developed signs for fulfilling the criteria of radiographic OA (according to Kellgren/Lawrence grade 1) in a 12-year period [30]. The motive for changing the inclusion criteria in this study was due to low patient inflow. The diagnostic accuracy of the ACR [40] clinical criteria for patients with early mild OA could have been too low. ACR clinical criteria seem to reflect later signs in advanced disease [65]. Future research would certainly benefit from specific criteria to detect early KOA to enhance the knowledge of early diagnosis and treatment in this stage of the disease.

One reason for the low patient flow could have been organisational, which involved both primary care centres and rehabilitation centres in primary care during the study period. The recruitment process was closed when no new participant was recruited for an entire year. Ongoing reorganisation was probably given priority at the clinics instead of recruiting study participants. Despite reorganisation, similar Swedish clinical trials have had similar problems with low patient flow when recruiting participants to their studies [66, 67].

Due to the low patient flow, the required sample size of 100 patients was not reached, which is a limitation of this study. Sixty-nine participants were included at baseline, which increases the risk of a type II error in the study. The dropout rate was 40% for the PT group and 32% for the physician group at the 12-month follow-up. The benefits of an analysis with mixed effect models is that participants with missing data can be used in the analysis as long as the missing data is missing-at-random. The missing value analysis for this study showed that the gender distribution, age range for the dropouts, and the reason for missing at follow-ups were similar in both groups (see Fig. 1). Mixed effect models handle the imbalanced data in available observations. Using mixed effect models is a strength of this study. The analysis is specifically designed for analysing data characterised by repeated measurements on the same individual [58]. The mixed effect models gave us a result showing how the primary assessment for patients with KOA affected their HrQoL 12 months after first assessment with consideration to possible confounders, which we cannot obtain from analyses with traditional statistical methods such as the Mann-Whitney U test.

A traditional randomised controlled trial with highly controlled treatments aim to test the true effect of a treatment, by ruling out placebo effects and extraneous effects (patient’s or healthcare provider’s knowledge or expectations of the treatment that could affect their behavior), and assuming that a patient group is homogenous. Compared to pragmatic trials, the generalizability for traditional randomised controlled trials are lower, since treatment outcomes would likely be affected by heterogeneous patient groups, placebo and extraneous effects which are present in the real clinic [59]. The pragmatic design is a strength of this study and the results have good external validity since the interventions have already been tested in a real clinical setting.

With this study, we showed that PTs and physicians did not differ as primary assessors for patients with suspected KOA, regarding HrQoL up to 12 months after patients consulted primary care. These results support previous findings showing that PTs could be used as the primary assessor for patients with musculoskeletal disorders. Our results imply a task shift in primary care, which would probably enhance access for patients with KOA to a better OA management including core treatment of patient education and exercise.

## Conclusions

In this study, we found no differences in HrQoL, when adjusted for pain and physical function, for patients with KOA when the healthcare process was initiated with a PT assessment compared to a physician assessment in primary care. Both assessments resulted in significantly higher HrQoL at the 12-month follow-up. The results imply that PTs and physicians in primary care are equally qualified as primary assessors.

## Additional file

Additional file 1: Tables of mixed effect models analysing EQ-5D-3L index and EQ-5D-3L VAS the first three months of the healthcare process. (DOCX 33 kb)

## Data Availability

The data sets generated and analysed during the current study are not publicly available due to the General Data Protection Regulation, which means that every participant’s data is confidential, and unauthorized persons have no access to the dataset, but are available from the corresponding author on reasonable request.

## Abbreviations

30CST: 30 seconds Chair Stand Test
ACR: American College of Rheumatology
BMI: Body mass index
CAT: Carina A Thorstensson (co-author)
CH: Chan-Mei Ho (corresponding author)
EQ-5D-3L index: EuroQol 5 dimensions 3 levels questionnaire index
EQ-5D-3L VAS: EuroQol 5 dimensions 3 levels questionnaire visual analogue scale
EQ-5D-5L: EuroQol 5 dimensions 5 levels questionnaire
EULAR: European League Against Rheumatism
KOA: Knee osteoarthritis
LN: Lena Nordeman (co-author)
n: Number
OA: Osteoarthritis
OARSI: Osteoarthritis research society international
PT: Physiotherapist/physiotherapy
SD: Standard deviation
VAS: Visual analogue scale
