# Supplementary material for: Physiotherapist as primary assessor for patients with suspected knee osteoarthritis in primary care—a randomised controlled pragmatic study
Source: BMC Musculoskelet Disord. 2019 Jul 13;20:329. doi: 10.1186/s12891-019-2690-1 (PMC6626628; doi:10.1186/s12891-019-2690-1)
Supplement: Supplementary file 1 — Tables of mixed effect models analysing EQ-5D-3L index and EQ-5D-3L VAS the first three months of the healthcare process. (DOCX 33 kb) [file 12891_2019_2690_MOESM1_ESM.docx]

**Additional file 1.** Table of Mixed effect models analysing first 3 months of the healthcare process.

| **Additional file 1a.** Mixed effect model analysis of EQ-5D-3L index – baseline to 3- month follow-up. | | | | | | | | |
| --- | --- | --- | --- | --- | --- | --- | --- | --- |
| **Model 1 (step 1)** | | **Model 1 with confounders (step 2)** | | | | | | **Model 2 (step 3, final)** |
| Group | 0.05* | 0.061 | 0. 059 | 0.053 | 0.083 | 0.30 | 0.15 | 0.42 |
| Time | 0.13 | 0.14 | 0.12 | 0.14 | 0.12 | 0.10 | 0.11 | 0.085 |
| Group x Time | 0.32 | 0.32 | 0.33 | 0.33 | 0.30 | 0.26 | 0.28 | 0.24 |
|  |  |  |  |  |  |  |  |  |
| Potential confounders | | **P-values for potential confounders when added one at a time** | | | | | |  |
|  | Age | 0.64 |  |  |  |  |  | N/A |
|  | Sex |  | 0.21 |  |  |  |  | N/A |
|  | BMI^a^ |  |  | 0.67 |  |  |  | N/A |
| Educational level^b^ | |  |  |  | 0.016 |  |  | 0.036 |
| Pain intensity^c^ | |  |  |  |  | <0.001 |  | <0.001 |
| Physical function^d^ | |  |  |  |  |  | 0.0082 | 0.32 |
|  |  |  |  |  |  |  |  |  |
| Presenting p-values from regression analyses using mixed effect models.  Model 1: Model included the variables Group, Time and Group x Time.  Model 1 with confounder: Confounders were added separately to Model 1. Confounding variables with p-values <0.2 were carried forward to the final model.  Model 2: Final model, adjusted for confounders (educational level, pain intensity and physical function).  Group: PT group resp. physician group.  Time: Measurements at baseline, 3-, 6- and 12-month follow-ups.  Group x Time: Statistical interaction of group and time.  ^a^Body Mass Index.  ^b^Educational level, dichotomized variables - primary and secondary or tertiary school.  ^c^Pain intensity, VAS 0-100 mm.  ^d^Physical function, 30 second Chair Stand Test.  *Statistically significant, p<0.05. | | | | | | | | |

| **Additional file 1b.** Mixed effect model analysis of EQ-5D-3L VAS – baseline to 3-month follow-up. | | | | | | | | |
| --- | --- | --- | --- | --- | --- | --- | --- | --- |
| **Model 1 (step 1)** | | **Model 1 with confounders (step 2)** | | | | | | **Model 2 (step 3, final)** |
| Group | 0.15 | 0.26 | 0.19 | 0.12 | 0.23 | 0.40 | 0.51 | 0.99 |
| Time | 0.21 | 0.23 | 0.19 | 0.17 | 0.21 | 0.19 | 0.16 | 0.14 |
| Group x Time | 0.72 | 0.70 | 0.75 | 0.67 | 0.71 | 0.68 | 0.64 | 0.55 |
|  |  |  |  |  |  |  |  |  |
| Potential confounders | | **P-values for potential confounders when added one at a time** | | | | | |  |
|  | Age | 0.027 |  |  |  |  |  | 0.010 |
|  | Sex |  | 0.016 |  |  |  |  | 0.56 |
| BMI^a^ | |  |  | 0.015 |  |  |  | 0.061 |
| Educational level^b^ | |  |  |  | 0.50 |  |  | N/A |
| Pain intensity^c^ | |  |  |  |  | 0.016 |  | 0.20 |
| Physical function^d^ | |  |  |  |  |  | <0.001 | <0.001 |
|  |  |  |  |  |  |  |  |  |
| Presenting p-values from regression analyses using mixed effect models.  Model 1: Model included the variables Group, Time and Group x Time.  Model 1 with confounder: Confounders were added separately to Model 1. Confounding variables with p-values <0.2 were carried forward to the final model.  Model 2: Final model, adjusted for confounders (age, sex, body mass index, pain intensity and physical function).  Group: PT group resp. physician group.  Time: Measurements at baseline, 3-, 6- and 12-month follow-ups.  Group x Time: Statistical interaction of group and time.  ^a^Body Mass Index.  ^b^Educational level, dichotomized variables - primary and secondary or tertiary school.  ^c^Pain intensity, VAS 0-100 mm.  ^d^Physical function, 30 second Chair Stand Test.  *Statistically significant, p<0.05. | | | | | | | | |
